# Development of microspheres for biomedical applications: a review

**DOI:** 10.1007/s40204-014-0033-8

**Published:** 2014-12-10

**Authors:** Kazi M. Zakir Hossain, Uresha Patel, Ifty Ahmed

**Affiliations:** Bioengineering and Advanced Materials Research Group, University of Nottingham, University Park, Nottingham, NG7 2RD UK

**Keywords:** Microspheres, Porous, Glasses, Ceramics, Polymers, Tissue engineering and regenerative medicine

## Abstract

An overview of microspheres manufactured for use in biomedical applications based on recent literature is presented in this review. Different types of glasses (i.e. silicate, borate, and phosphates), ceramics and polymer-based microspheres (both natural and synthetic) in the form of porous , non-porous and hollow structures that are either already in use or are currently being investigated within the biomedical area are discussed. The advantages of using microspheres in applications such as drug delivery, bone tissue engineering and regeneration, absorption and desorption of substances, kinetic release of the loaded drug components are also presented. This review also reports on the preparation and characterisation methodologies used for the manufacture of these microspheres. Finally, a brief summary of the existing challenges associated with processing these microspheres which requires further research and development are presented.

## Introduction

The development of microspheres fabricated from biopolymers (Freiberg and Zhu 2004), bioactive glasses (Lakhkar et al. 2012) and ceramics (Bohner et al. 2013) is an ongoing challenge for many researchers across the globe. Microspheres possess several advantages for use in biomedical applications over other particle geometries; for example, they can be manufactured to have a uniform size and shape which can improve delivery of the spheres to the specific target site, a larger surface area allowing for sufficient therapeutic coatings and an increase in degradation rate and ion release and can in some cases be engineered to be porous or hollow, allowing for encapsulation of other biomedically relevant components (Cai et al. 2013; Freiberg and Zhu 2004; Li et al. 2010). Porous microspheres can be fabricated with either external or internal porosity, or even a combination of both, as well as with or without interconnectivity for cell attachment and spreading over the available surface area (Chen et al. 2011). Microspheres containing tailored porosity exhibit greater surface area, lower mass density, superior cell attachment, cell proliferation, drug absorption and drug release kinetics compared to bulk microspheres (Cai et al. 2013). In addition, these microspheres can be fabricated as stand-alone products or assembled into three-dimensional (3D) porous scaffolds (Cai et al. 2013; Li et al. 2010; Perez et al. 2011).

Specific applications have been designated for porous microspheres based on the composition of these materials as well as the pore structures (e.g. level of porosity, pore size, surface area, interconnectivity, etc.). For example, polymer-based porous microspheres have been extensively investigated for drug release and as other biological component (proteins, cells, growth factors) delivery vehicles (Cai et al. 2013; Freiberg and Zhu 2004), whereas ceramic (Komlev et al. 2002; Liu 1996) and glass (Lakhkar et al. 2012) based microspheres have been mainly investigated for bone tissue regeneration (Choi et al. 2012), radionuclide therapy (Sene et al. 2008), dental and orthopaedic applications (Bohner et al. 2013).

This review aims to provide a general overview of microspheres used in the biomedical sector, focusing on the manufacturing methodologies of porous and non-porous microsphere production, utilising different types of biomaterials.

## Manufacture and characterisation of microspheres

### Glass microspheres

Glass materials for biomedical applications have long been investigated for their use in the repair, restoration and regeneration of tissue within the human body. Larry Hench revolutionised the use of glassy materials for biomedical applications since the discovery of Bioglass^®^ during the late 1960s (otherwise known as 45S5). There are now three major glass types under investigation for biomedical applications, which include the conventional silicate based glasses, phosphate-based glasses and borate-based glasses (Hench 2006; Jones 2013; Rahaman et al. 2011). In terms of the manufacture of these glasses, many studies have focused on analysing them in bulk form, rods, discs and more recently fibres (Abou Neel et al. 2007; Abou Neel et al. 2009b; Ahmed et al. 2004a, b; Hossain et al. 2014b; Knowles 2003). However, uses of these materials in microsphere form are now receiving much attention.

**Fig. 1:**
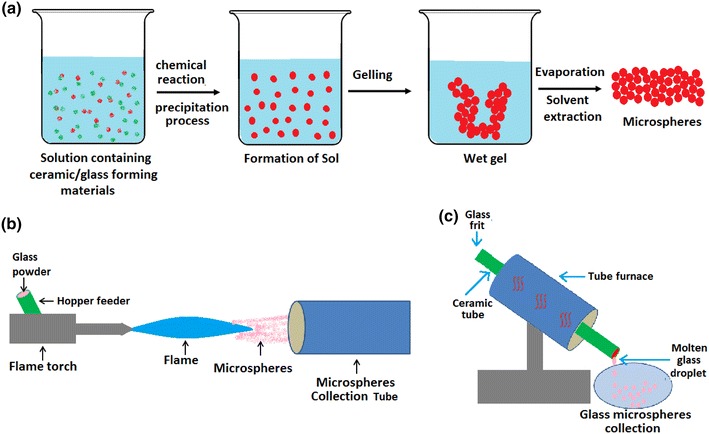
Scheme of production of glass microspheres via **a** Sol–gel, **b** flame spheroidisation and **c** tube furnace methods

Methods of creating glass spheres have included dropping crushed glass particles down a vertical tube furnace (Fu et al. 2010), pouring molten glass onto stainless steel plates to create droplets (Huang et al. 2009), sol–gel method and spray drying of sols (Todea et al. 2013) and via the flame spheroidisation process (Lakhkar et al. 2012) (see Fig. 1). Sols of varying glass compositions (such as aluminosilicate) are usually produced by chemical precipitation method and then the microspheres are formed either by spray drying of the sols or via a solvent evaporation process (Todea et al. 2013). On the other hand, vertical tube furnaces and flame spheroidisation processes involve grinding the desired composition of the bulk glass into particles. Usually, if a desired dimension is required the particles may be separated into varying size ranges via sieving. The crushed particles are then transformed into spherical shapes either by being passed through a vertical tube furnace (Day et al. 2003; Fu et al. 2010) or being fed into a hot flame where the high temperatures and surface tension cause the glass particles to re-melt and form spheres (Lakhkar et al. 2012).

The flame spheroidisation technique is a relatively fast, inexpensive process which can easily be scaled-up for commercialisation purposes. However, for manufacture of larger microspheres the tube furnace process usually yields better results. There are several parameters of the flame spheroidisation technique which can all affect the outcome of the sphere size and shape; particle separation before entering the flame is a key criterion to obtain dispersed uniform spheres; residence time in the flame is also an important factor as larger particles will require a longer residence time for the glass to spheroidise. The flame temperature can also determine the dimensions of the microspheres and this temperature is generally controlled by the fuel used. Several studies have utilised varying gases to create a flame including propane/oxygen, acetylene/oxygen, petrol/oxygen (Martinelli et al. 2010) and natural gas/air flames (Conzone et al. 2002; Fu et al. 2010; Lakhkar et al. 2012; Wang et al. 2006).

#### Borate-based glass microspheres

**Fig. 2:**
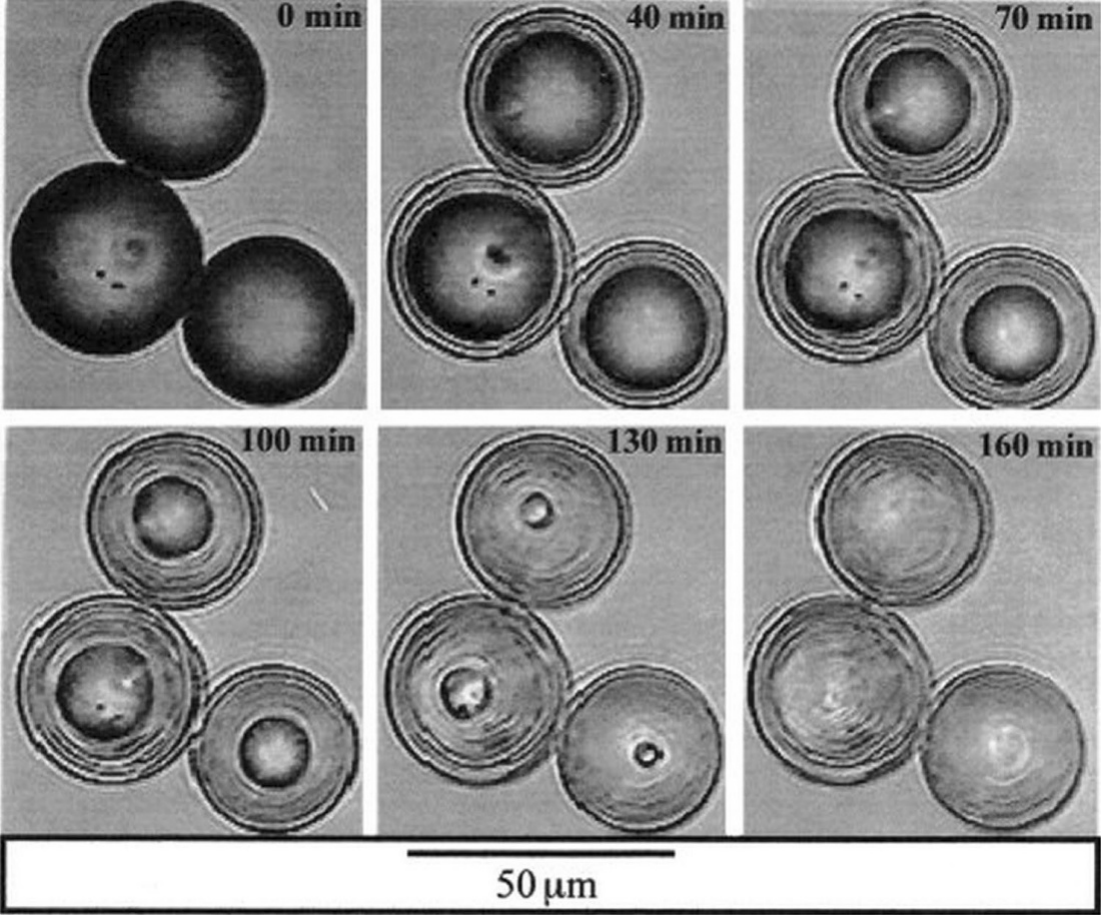
Real-time video microscopy image showing non-uniform reaction of Dysprosium Lithium-Borate glass microspheres in PBS solution at 37 °C (Conzone et al. 2004)

Borate-based glass microspheres have been of particular interest for use as biodegradable radiation delivery vehicles, in particular Dysprosium lithium-borate microspheres for the treatment of rheumatoid arthritis (Conzone et al. 2002, 2004). The way in which these spherical vehicles were previously processed included initial fabrication of glass microspheres using non-radioactive materials via the flame spheroidisation technique. These microspheres made for an ideal candidate for radiation synovectomy due to their uniform size and shape, as well as their post processing capability for generating radioactive microspheres. In order to yield these microspheres radioactive, the isotopes which were chemically incorporated into the structure of the glass were neutron activated, before being injected into the site of interest. Microsphere size was also an important factor to consider when fabricating delivery vehicles to accommodate their constituents, as well as yielding suitable dimensions which could be delivered and retained at the site of interest. Conzone et al. (2002, 2004) fabricated microspheres with a diameter range between 5 and 15 µm to prevent particulate leakage during radiation synovectomy for the treatment of rheumatoid arthritis. Although these microspheres exhibited a uniform shape, their reaction behaviour in simulated synovial fluid (SSF) was far from uniform. It was seen that the soluble components of the glass composition (lithium and Boron) were discharged into the SFF, whereas the insoluble dysprosium remained chemically intact in the reacted microspheres resulting in a porous dysprosium phosphate-rich product. On initial submersion into SSF, the non-uniform reaction caused the formation of a reaction layer which later linearly propagated towards the centre of the microsphere, resulting in ~80 % weight loss after 64 days without changing their size and shape. From this it was found that this non-uniform behaviour was not an outcome of spheroidisation, but rather due to the soluble and insoluble constituents of the glass, as identical results were observed for non-spherodised particles (Conzone et al. 2004). Similar non-uniform reaction of these glass microspheres within PBS solution at 37 °C suggested that the microspheres had completely reacted inside 160 min to form a dysprosium phosphate-rich reactive product (see Fig. 2) (Conzone et al. 2004).

Following on from these findings, the resultant amorphous, porous reaction products were exploited and a “novel” chemical process was described, yielding porous microspheres with the same size and shape as the starting product, however, of a different composition. The non-uniform reaction process of dysprosium lithium-borate glass microspheres in phosphate-containing solutions at 37 °C presented porous microspheres with a specific surface area of around 200 m^2^/g, pore volume of 0.2–0.4 cm^3^/g and pore diameters of around 30 nm (Conzone and Day 2009).

**Fig. 3:**
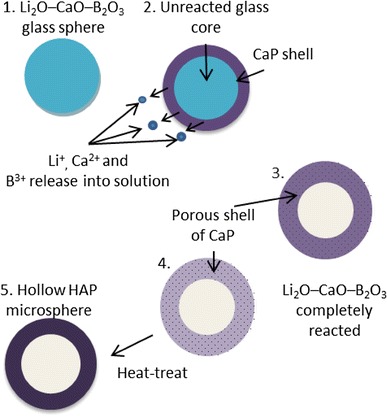
Schematic illustration showing mechanism for production of hollow, porous HAP microspheres (Wang et al. 2007)

Alternate two-stage processes involving borate-based glass microspheres have included the conversion of bulk Li_2_O–CaO–B_2_O_3_ solid microspheres produced via flame spheroidisation into hollow hydroxyapatite (HAP) microspheres by reacting the solid microspheres in a buffer solution (0.25 M K_2_HPO_4_) (Huang et al. 2009; Wang et al. 2007, 2006). Briefly, Li_2_O–CaO–B_2_O_3_ glass microspheres reacted with K_2_HPO_4_ solution, resulting initially in heterogeneous precipitation of calcium phosphate after a reaction period of 5 days. Following this, subsequent heat treatment (at 600 °C for 4 h) of these amorphous calcium phosphate hollow shells resulted in crystallised and porous HAP microspheres (Huang et al. 2009; Wang et al. 2007, 2006). The mechanism for the production of these hollow microspheres is illustrated in Fig. 3.

Post heat treatment also resulted in improved strength of these microspheres; however an increase in brittleness was also observed. A study conducted by Huang et al. (2009) measured the strength of these hollow HA microspheres comparing the as prepared microspheres to the heat-treated microspheres. It was found that the large surface area of the as prepared microspheres (135 m^2^/g) was drastically reduced after heat treatment for 8 h at 600 °C, and on heat treating at 800 °C, the surface area reduced by a factor of more than 50× to 2.6 m^2^/g and compressive strength increased to 35 ± 8 MPa as opposed to 1.6 ± 0.6 MPa for the as-prepared hollow microspheres. It was suggested that the geometry of the spheres was more likely to effect the strength rather than the size, since experiments conducted on microspheres with diameters of approximately 500 and 800 µm showed no difference in these properties, confirming that the compressive strength of structurally and compositionally homogenous porous spheres are independent of their size (Huang et al. 2009). An even greater specific surface area of 145 ± 5 m^2^/g was achieved by Fu et al. (2010) when reacting the glass microspheres with 0.25 M K_2_HPO_4_ at a reaction temperature of 60 °C, as these parameters provided a high concentration of phosphate ions and a beneficial temperature, causing finer particle sizes of HA to form, resulting in higher specific surface area. The same study also found that reducing the reaction temperature (25 °C) and the concentration of K_2_HPO_4_ (0.02 M) resulted in larger ratio between the hollow core diameter to the external diameter of the microspheres, thought to be due to a more efficient packing of the fine HA particles.

Borate glasses are an ideal material to fabricate these hollow microspheres due to their low network connectivity and ease of hydrolysis in acidic or basic solutions. Scanning electron microscopy (SEM) images have shown these microspheres to consist of multiple porous layers which make up the shell wall of the hollow microspheres. In general it was found that the outer layers of the shells were smooth and less porous than the inner layer. The most effectual variables on pore size have been found to be K_2_HPO_4_ concentration and reaction temperature with low solution concentrations (0.02 M) and high reaction temperatures (60 °C) resulting in the smallest pore sizes (outer shell wall pore size of ~10 nm). This reduction in pore size and formation of multiple layers are likely to occur due to densification of the HA shell and separation during the conversion reaction (Fu et al. 2010; Huang et al. 2009).

#### Silicate-based glass microspheres

**Fig. 4:**
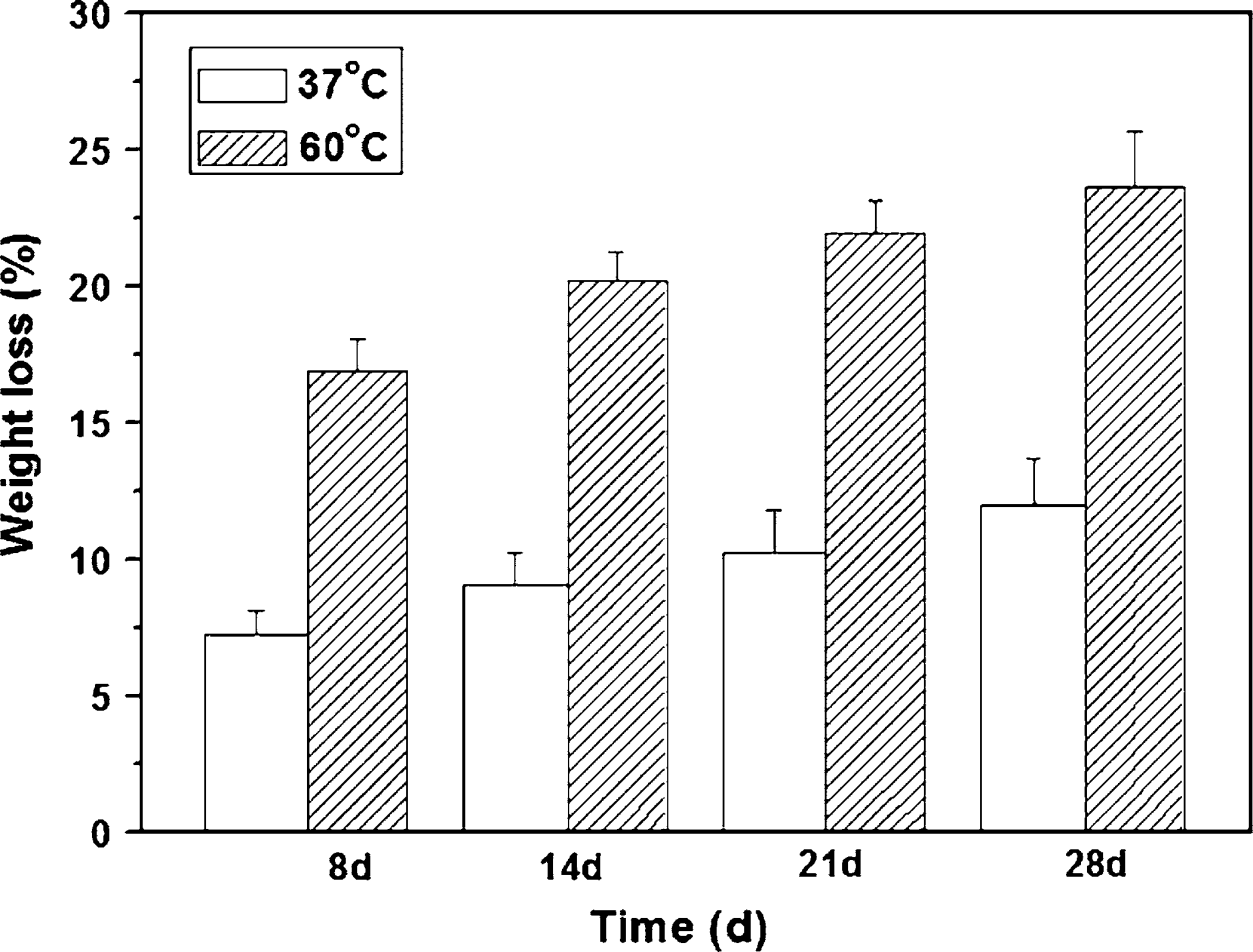
Weight loss of 45S5 glass–ceramic microspheres observed over a period of 4 weeks in 1.0 M K_2_HPO_4_ solution (Fu et al. 2012)

Silicate-based bioglass, glass–ceramics microspheres (Fu et al. 2012) and silica nanospheres (Stöber et al. 1968) have been recently investigated for biomedical application. A glass–ceramic phase can be defined as material where one or several crystal phases are embedded in a glassy matrix. The narrow window between the glass thermal transition temperature (*T*_g_) and its onset of crystallisation can lead to the conversion of a glass–ceramic material when attempting thermal processing such as flame spheroidisation. A study by Fu et al. (2012) evaluated the conversion of silicate-based glass–ceramic microspheres (designated as 45S5c) to a HA-like material. 45S5c glass–ceramic microspheres with a diameter between 75 and 150 µm were fabricated using 45S5 glass powder via flame spheroidisation technique and immersed in 0.01 and 1.0 M of K_2_HPO_4_ solution for a long-term period (10 years) at room temperature. These results were compared to microspheres immersed in the same concentration of K_2_HPO_4_ solution for a shorter period of 4 weeks (Fig. 4). The results showed that even after 10 years, conversion to a calcium phosphate material was still incomplete. One possible reason for this was suggested to be due to the presence of a combeite crystalline phase (Na_2_O–2CaO–3SiO_2_) observed after the spheroidisation process of 45S5 glass. However, using a 3D diffusion model they predicted a time of approximately 45 years for the full conversion of 45S5 glass–ceramic microspheres in K_2_HPO_4_ solution (at 37 °C) to HA-like materials, thus suggesting that the unconverted glass ceramic could remain in the body for very long periods (Fu et al. 2012).

Other glass–ceramic microspheres fabricated include aluminium iron silicate glasses, with a main crystalline phase of magnetite, which were investigated for use in thermotherapy to treat liver cancers (Martinelli et al. 2010). Particle sizes of 38–63 µm were spheroidised using flame to manufacture microspheres; however, the resultant size distribution of the microspheres produced was greater than the original particle size, which surpassed 100 µm for some spheres.

Other methods used to create microspheres have included the sol–gel method via the Stöber process investigated by Liu et al. (2012). Hydrolysis and polycondensation of tetraethoxysilane ethanol solution (TEOS) have been shown to produce monodispersed silica microspheres (0.3 µm) due to repulsive forces encountered via the negative charges created under alkaline conditions. On addition of aluminium nitrate (Al(NO_3_)_3_·H_2_O) and silver nitrate (AgNO_3_) dissolved in MeOEtOH, amorphous microspheres (0.4 µm) coated in finer particles resulted, leading to aggregation of the samples. Although subsequent heat treatment at 1000 °C formed larger microspheres (8.8–10.1 µm) with smoother surfaces, aggregation of the particles occurred and it was found that with increasing Al(NO_3_)_3_·H_2_O and AgNO_3_, aggregation increased linearly. The antibacterial agent releases, i.e. silver ions, were only effective during initial submersion of these microspheres in ultrapure water. This was suggesting that silver nitrate incorporation occurred only on the surface of the spheres, creating a sort of short-term antibacterial shell surrounding a silica core (Kawashita et al. 2003). An alternate method of adding aluminium tri-isopropoxide (Al(OC_3_H_7_)_3_) powder to a partially hydrolysed TEOS to polycondense the solution to form Si–O–Al bonds was used. Silver ions were subsequently added to the ATIP/TEOS mixture in a solution of ammonia and silver nitrate and a centrifuge was used to separate solid products isolated from the solution. The resultant monodispersed microspheres had diameters ranging from 0.4 to 0.6 µm which did not change following subsequent heat treatment. Furthermore aggregation of the microspheres was not observed after application of heat, and release rates of silver ions in water were a lot more gradual than before. This more controlled release of silver ions was due to the fact that during fabrication of the microspheres, the silver ions enter the SiO_4_ network accompanying the aluminium ions in the form of [AlO_4_]^−^ Ag^+^, and ion exchange with H_3_O^+^ in the water slowly released Ag^+^ ions from the microspheres. These alternate silver-doped microspheres have vast potential for use as antibacterial materials.

#### Phosphate-based glass microspheres

**Fig. 5:**
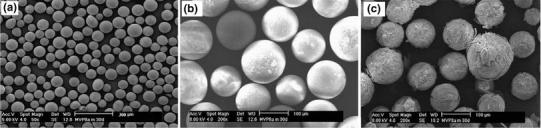
SEM image of **a** phosphate glass microspheres produced using flame spheroidisation process and **b** microspheres after 21 days of immersion in simulated body fluid (SBF) at 37 °C **a** MVP9c and **b** MVP3 microspheres (Sene et al. 2008)

The use of phosphate-based glasses (PBGs) for biomedical applications has seen a huge increase in interest in recent years which is still growing. This is mainly due to the desirable properties imparted by these glasses; which include ease of tailoring degradation profiles by simply altering their composition, their cytocompability and varying geometries that have been produced including fibres (Hossain et al. 2014b; Knowles 2003). Fabricating PBGs into microspheres has also very recently been reported (Lakhkar et al. 2012; Sene et al. 2008). Sene et al. (2008) produced amorphous phosphate glass microspheres of varying composition of P_2_O_5_, Al_2_O_3_, SiO_2_ and MgO (P_2_O_5_ content ranging from 40 to 60 wt % using flame (oxygen/petrol) spheroidisation process (see Fig. 5a).

They also investigated the degradation of these microspheres in simulated body fluid (SBF) for 21 days at 37 °C and found that the microspheres containing 32.6 at. wt % P content (MVP9c) was more stable to SBF as less precipitation could be seen on their surface compared to higher P content microspheres (Please see Fig. 5b, c).

**Fig. 6:**
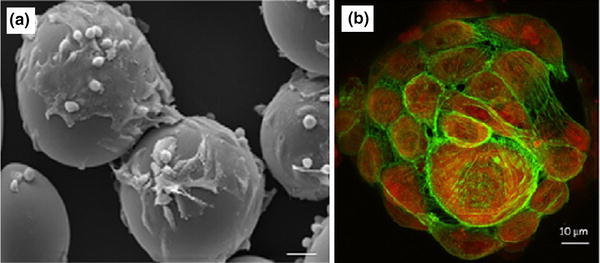
**a** SEM, and **b** scanning laser confocal microscopy (SLCM) images of titanium phosphate glass microspheres cultured with MG63 cells on day 7. *Scale bar* of SEM image represents 25 μ (Lakhkar et al. 2012)

Recently Lakhkar et al. (2012) produced titanium-doped phosphate glass microspheres also utilising the flame spheroidisation process. Microspheres were produced in the range of 63–106 µm and they found that producing microspheres below 30 µm was difficult as the particles would agglomerate in both the feed apparatus as well as in the flame. The larger particle size ranges were also unsuccessful in creating spheres as a longer residence time within the flame was required. They also investigated the structural characterisation and suggested that these microspheres were comparable to glasses of the same composition in other forms, such as powders and discs (Abou Neel et al. 2008, 2007, 2009b). As expected, the degradation profile of the microspheres behaved in an exponential manner compared to irregular shaped glasses of the same composition (Abou Neel et al. 2008, 2009a; Abou Neel and Knowles 2008), due to the increase in surface area of the microspheres. Furthermore, it was also shown that the titanium phosphate glass microspheres supported favourable MG63 osteoblastic cell attachment and proliferation on their surface (Lakhkar et al. 2012), as seen in Fig. 6. Both the SEM and scanning laser confocal microscopy (SLCM) images showed the microspheres were covered with a number of cells and some cells appeared to join neighbouring microspheres by means of their bioactivity.

### Ceramic microspheres

Calcium phosphate (CaP) based ceramic microspheres has become a common interest to researchers, particularly hydroxyapatite (HA) (Abou Neel et al. 2008, 2009a; Abou Neel and Knowles 2008; Abou Neel et al. 2007, 2009b; Ahmed et al. 2004a, b), and β-tricalcium phosphate (β-TCP) (Akamatsu et al. 2010; Athanasiou et al. 1998; Auras et al. 2003; Baldwin et al. 2010; Bergquist et al. 1972; Berkland 2004) for their use in orthopaedics, dentistry and the pharmaceutical sectors due to their excellent biocompatibility, osteoconductivity and adequate mechanical properties. These materials tend to be used alone or in combination with different polymer phases. The advantages associated with the use of ceramic microspheres include implantation via a minimally invasive route and they can provide mechanical support to the target site of application. Despite these advantages, these ceramic materials are associated with high brittleness and slow resorption rates compared to glass microspheres. In addition, the spherical shape of ceramic particles are considered to be more suitable for bone defect filling applications due to their packing and predictable flow characteristics during injection, compared to irregular shaped micro-particles (Bohner et al. 2013).

**Fig. 7:**
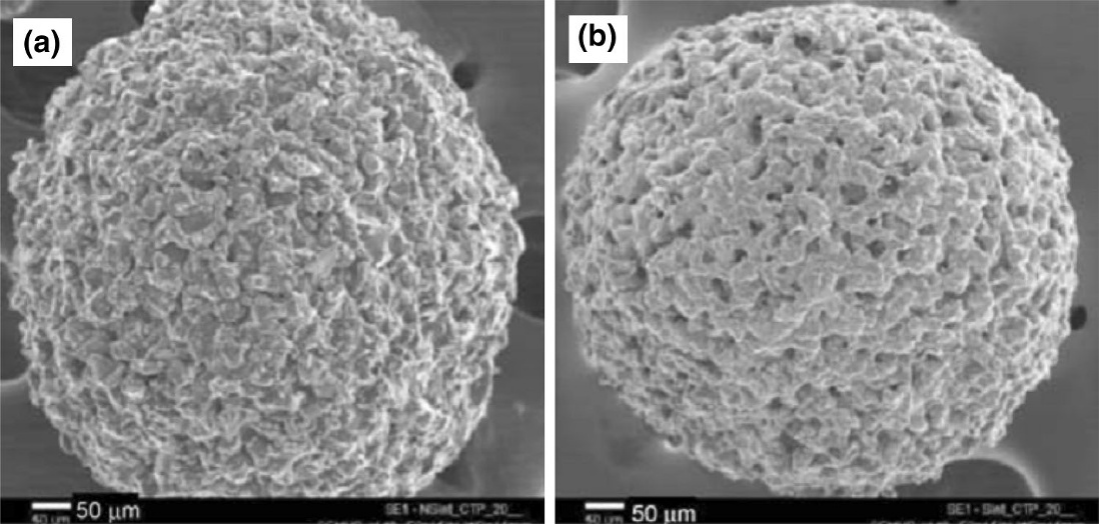
SEM images of calcium-titanium-phosphate (CTP) microspheres **a** Non-sintered CTP microspheres and **b** sintered CTP microsphere Ribeiro et al. (2006)

Bohner et al. (2013) highlighted the synthesis and application of ceramic microspheres in dental and orthopaedic applications in a recent review. Various methods employed for the production of spherical particles with a broad range of properties according to the starting materials (such as powders, slurries, pastes and solutions) and the dispersion phases (gas, solution, and solid). Other factors include the dispersion apparatus (syringe needles, spray nozzles, sieves, stirrers, propellers), and consolidation methods such as flame spraying (Cho et al. 2010), freeze drying (Hong et al. 2011), gelling (Paul and Sharma 1999) and chemical precipitation (Qiu et al. 2008) were reviewed. Ribeiro et al. (2006) manufactured porous ceramic microspheres with interconnected porous network by mixing calcium–titanium–phosphate (CTP) and HA with alginate solution using a droplet extrusion method followed by Ca^2+^ induced gelation and subsequent sintering to burn-off the polymer (see Fig. 7). The ratio of ceramic phase and polymer solution was a critical parameter to alter the size distribution of the microspheres produced. For example, microspheres with average diameters of 513 ± 24 and 602 ± 28 µm were reported using a CTP ceramic-to-polymer ratio of 10/3 and 20/3, respectively, whereas with HA the average diameters found were 429 ± 46 and 632 ± 40 µm for the same formulation.

Paul and Sharma (1999) developed porous HA microspheres by mixing HA particles with chitosan solution followed by glutaraldehyde addition. This process induced hardening leading to the formation of a spherical shape. The chitosan bonded microspheres produced were then heated at 500 °C for 3 h to burn off the organic matrices and finally sintered at 1,100 °C for 1 h to obtain a porous structure.

**Fig. 8:**
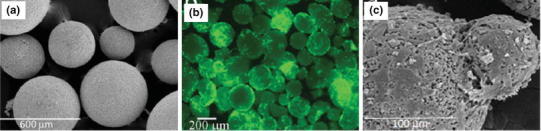
**a** SEM image of 5 % GEL/OF 900, **b** Morphology of Saos-2 cells on gelatin/hydroxyapatite microspheres and **c** after 14 days of culture Perez et al. (2011)

Perez et al. (2011) investigated porous HA and gelatin/HA microspheres (pore sizes ranging between 0.5 and 5 μ) obtained through a water-in-oil emulsion of calcium phosphate cement (CPC), where the setting reaction of the CPC influenced consolidation of the microspheres. The sphericity and size distribution of the microspheres were improved via incorporation of gelatin with the cement as presented in Fig. 8a. They suggested that cell adhesion (Saos-2 cells) and proliferation (see Fig. 8b, c) were significantly improved in the hybrid gelatin/HA microspheres as compared to the control HA microspheres.

Recently, Sui et al. (2013) developed a simple and inexpensive chemical transformation process to synthesise mesoporous calcium phosphate microspheres (CPMs). At first they produced calcium carbonate microspheres (CCMs) by reacting calcium chloride and sodium carbonate solutions at room temperatures which was obtained as a precipitated product. They then converted the CCMs into mesoporous CPMs by reacting with ammonium hydrogen phosphate solution. These CPMs were stable in aqueous media and had higher specific surface area compared to the CaCO_3_ microspheres. In addition, CPMs showed impressive encapsulation efficiency (40 % loading efficiency) with positively charged biomacromolecules such as carboxymethyl chitosan and doxorubicin. Calcium phosphate hollow bioceramic micorspheres with nano-sized pores were produced by Kawanobe et al. (2010) via a salt-assisted ultrasonic spray-pyrolysis technique and investigated vancomycin drug release profiles in physiological saline media at 37 °C for osteomyelitis treatment. They suggested that the microspheres showed two-step drug release behaviour: from the surface of the microspheres drug release was observed during the first 3 h and from inside the microspheres due to nano-size pores over 7–9 h.

Though a vast amount of research has been done on calcium phosphate ceramic microspheres for dental and orthopaedic applications [highlighted in the recent review by Bohner et al. (2013)] there still remain some issues related to these ceramic materials such as high cost, time-consuming lengthy or complicated manufacturing processes, brittleness and slow resorption rates.

### Polymer-based microspheres

**Fig. 9:**
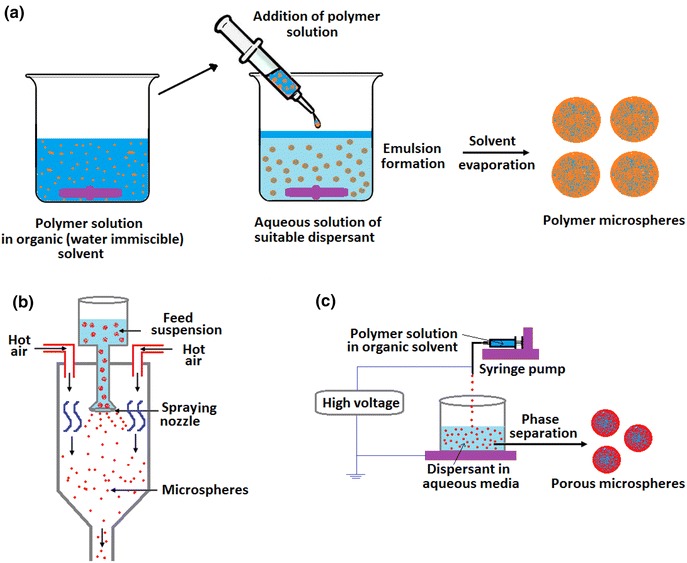
Typical schemes of production of polymer microspheres employing various methods **a** emulsion-solvent evaporation, **b** sol-spray drying and **c** electro-spinning processes

Polymer-based microspheres have received considerable attention in recent years due to their potential controlled drug release characteristics either by leaching the drug components from the polymer or by degradation of the polymer matrix (Edlund and Albertsson 2002; Kohane et al. 2006). As such, selection of biodegradable carrier matrices (either synthetic or natural) used for microsphere production is an important factor for delivery of therapeutic agents (Jung et al. 2000). Most natural polymers such as proteins (Bergquist et al. 1972; Han et al. 2008), collagen (Hong et al. 2012; Nagai et al. 2010; Yao et al. 2013), chitosan (Akamatsu et al. 2010; Maeng et al. 2010; Oliveira et al. 2005; Torres et al. 2007) and alginate (Chan et al. 2002; Eiselt et al. 2000; Lemoine et al. 1998; Mofidi et al. 2000; Ribeiro et al. 2005) degrade by enzymatic activity, whereas synthetic polymers such as polylactic acid (PLA), polycaprolactone (PCL), polyglycolic acid (PGA) and polylactic-co-glycolic acid (PLGA) undergo hydrolytic degradation in the body (Jung et al. 2002). Several methods have also been investigated to produce polymer microspheres for biomedical and pharmaceutical interests (see Fig. 9), such as emulsion-solvent evaporation (Wang et al. 2002), spray drying (Oliveira et al. 2005; Wang et al. 2004), electro-spinning (Bock et al. 2011; Maeng et al. 2010), gelation followed by emulsification (Chan et al. 2002; Ribeiro et al. 2005), suspension polymerisation (Bergquist et al. 1972), ultrasonication (Han et al. 2008) and phase separation (Zhao et al. 2004), which will be discussed further in the following sections.

#### PLA microspheres

PLA is one of the most common bioresorbable polymers used in the biomedical sector due to its degradation rate, good mechanical properties and availability in different lactide contents (i.e. L/D ratio) (Athanasiou et al. 1998; Waris et al. 2004). In addition, thermoplastic PLA can be formed into various architectural forms including films (Auras et al. 2003; Hossain et al. 2012), scaffolds (Chung et al. 2011; Montjovent et al. 2005), fibres (Hossain et al. 2014a, c; Leenslag and Pennings 1987), rods (Felfel et al. 2011) and microspheres (Ehtezazi and Washington 2000; Izumikawa et al. 1991; Ruan and Feng 2003; Zielhuis et al. 2006). It has also been processed via solvent (Chung et al. 2001) and emulsion-solvent (Hong et al. 2005; Ruan and Feng 2003) evaporation processes to produce microsphere structures with varying morphologies. Izumikawa et al. (1991) investigated progesterone drug-loaded poly (l-lactide) (PLLA) microspheres (diameter 44–88 µm) prepared via a solvent evaporation method for controlled drug release applications. They dispersed PLA/methylene chloride solution into 1 wt % of gelatine/water solution at constant stirring, followed by removal of the volatile solvent using varying pressures to control the crystallinity of the polymer microspheres produced. It was reported that removal of volatile solvent at atmospheric pressure lead to formation of PLA microspheres with a crystalline structure, whereas at reduced pressures (i.e. 200 mm Hg), the escaping solvent produced microspheres with amorphous polymer matrices. They further suggested that the crystalline PLLA microspheres had a rough surface with large surface areas which revealed rapid drug release profiles (around 90 % after 145 h) compared to the smooth amorphous PLLA microspheres (which revealed drug release rates of 40 % at 145 h).

Antineoplastic drug paclitaxel-loaded poly(lactic acid)–poly(ethylene glycol)–poly(lactic acid) (PLA–PEG–PLA) microspheres of various compositions were produced by Ruan and Feng (2003) employing the oil-in-water single-emulsion solvent extraction/evaporation method. They suggested that incorporation of a water-soluble solvent (acetone) in the organic solvent (dichloromethane) phase during microsphere fabrication, along with the presence of a hydrophilic PEG segment within the hydrophobic PLA increased porosity of the microspheres and also facilitated faster paclitaxel release. For example, a (49.6 %) sustained release of paclitaxel over 1 month was achieved for the PLA–PEG–PLA microspheres compared to the control PLGA (L/G ratio = 50/50) microspheres which only released around 22 % of the drug over the same time period.

**Fig. 10:**
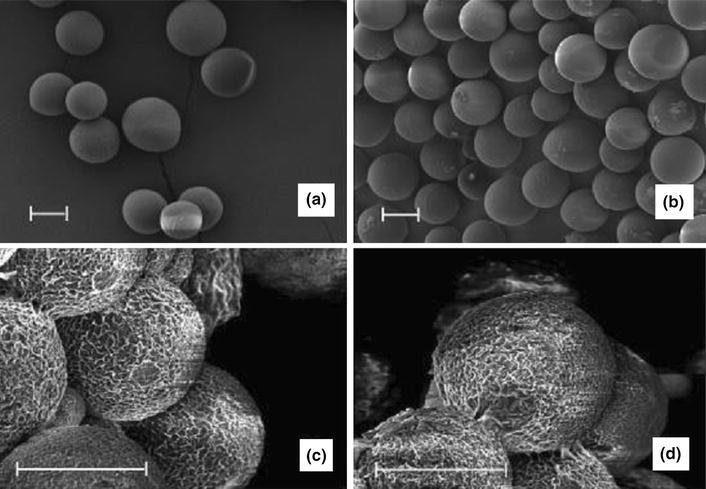
SEM images of **a** PLLA microspheres, **b** gamma-irradiated PLLA microspheres, **c** Ho-PLLA microspheres and **d** gamma-irradiated Ho-PLLA microspheres after 52 weeks of incubation in phosphate buffer. *Scale bars* represent 20 μ (Zielhuis et al. 2006)

In vitro degradation analysis over a 52-week period of holmium-loaded PLLA (Ho-PLLA) microspheres (before and after neutron or gamma irradiation) was investigated by Zielhuis et al. (2006). PLLA microspheres (diameter ranging from 20 to 50 μ) were produced by dissolving PLLA in chloroform and then dispersing the solution of organic solvent into an aqueous solution of PVA (2 wt %) as presented in Fig. 10a. They reported that incorporation of Ho within PLLA and neutron irradiation accelerated the degradation profile of the microspheres, releasing a significant portion of disintegrated fragments consisting of insoluble holmium lactate microcrystals (see Fig. 10c, d) compared to the other formulations investigated.

**Fig. 11:**
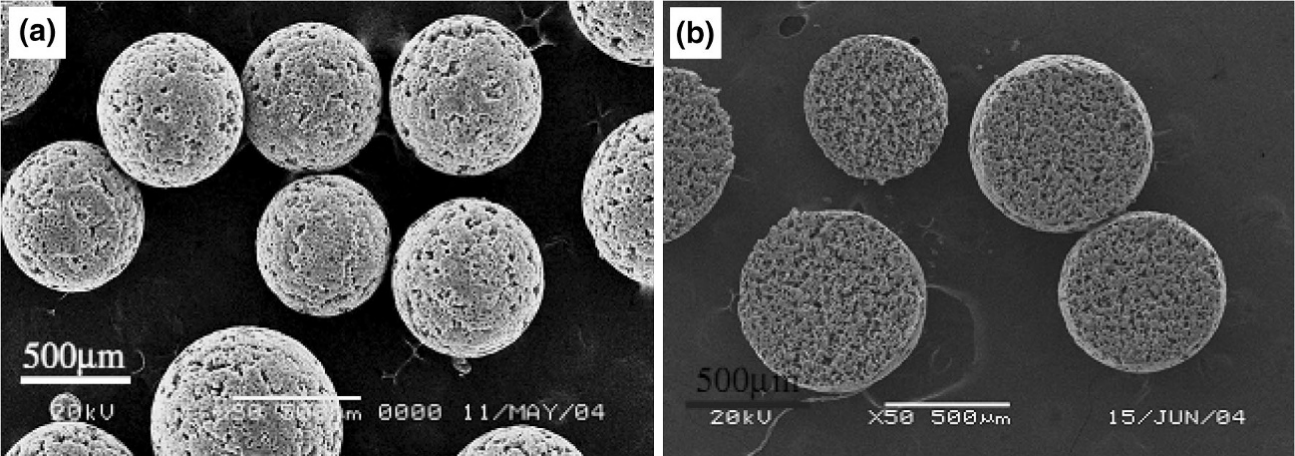
SEM images of PLA microspheres produced using emulsion-solvent evaporation process **a** surface morphology, and **b** internal cross-section image (Hong et al. 2005)

PLA microspheres with interconnected porosity (see Fig. 11) were fabricated by an emulsion-solvent evaporation method based on solution induced phase separation by Hong et al. (2005). They suggested that the processing conditions such as organic solvent/aqueous solvent ratio, PLA concentration, flow, stirring rate and dispersant (such as, polyvinyl alcohol) concentration all had an important influence on the size distribution and pattern of pores within the microspheres produced. For instance, a comparatively larger pore size had been achieved at a slower stirring rate, lower organic solvent/aqueous solvent ratio and with a lower PLA concentration due to longer coalescence time.

#### PCL microspheres

**Fig. 12:**
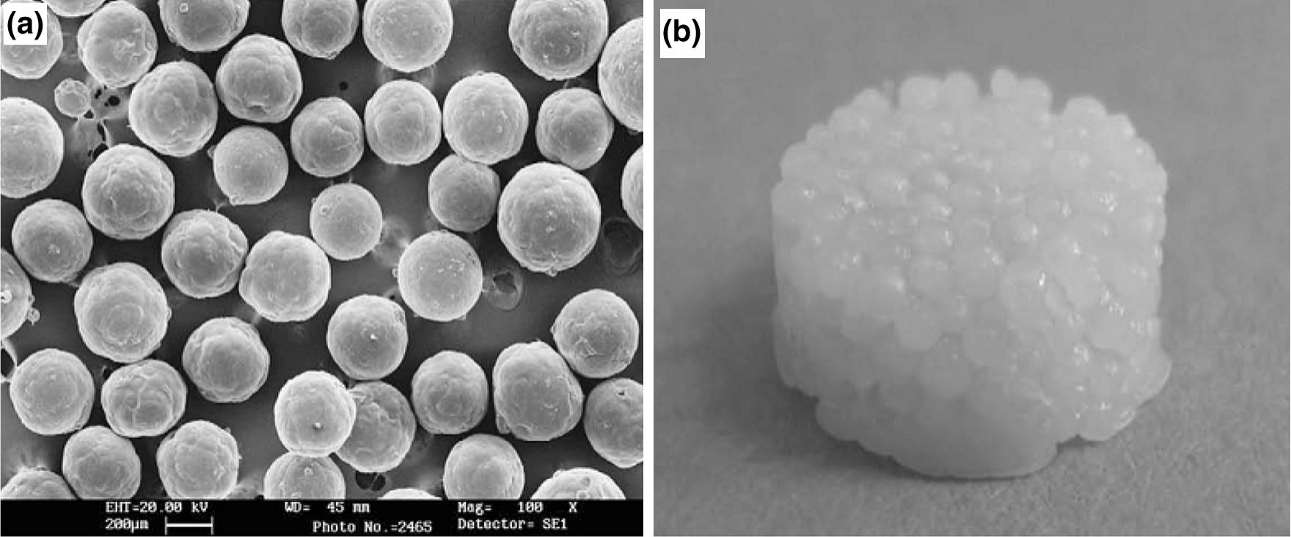
SEM micrographs of **a** PCL microspheres, obtained by single emulsion, **b** photograph of PCL microspheres sintered scaffold (Luciani et al. 2008)

A number of synthetic polymers have been investigated for biomedical and tissue engineering applications; among these, poly(ɛ-caprolactone) (PCL) is one of the most widely used bioresorbable polymers. Like PLA, PCL can also be fabricated into microspheres via several methods, such as emulsion-solvent evaporation (Luciani et al. 2008), electro-spinning (Bock et al. 2011) and melt moulding (Lin et al. 1999) processes. For example, (Luciani et al. 2008) produced protein (Bovine serum albumin)-activated PCL microspheres by double-emulsion (using dichloromethane solvent and aqueous PVA solution) and protein-free PCL microspheres via a single-emulsion technique (Fig. 12a). In addition, PCL microspheres were sintered at 60 °C for 1 h for the fabrication of bioactive scaffolds as seen in Fig. 12b. It was also reported that protein-loaded microspheres were successfully included within the scaffold which provided a sustained release of the protein.

**Fig. 13:**
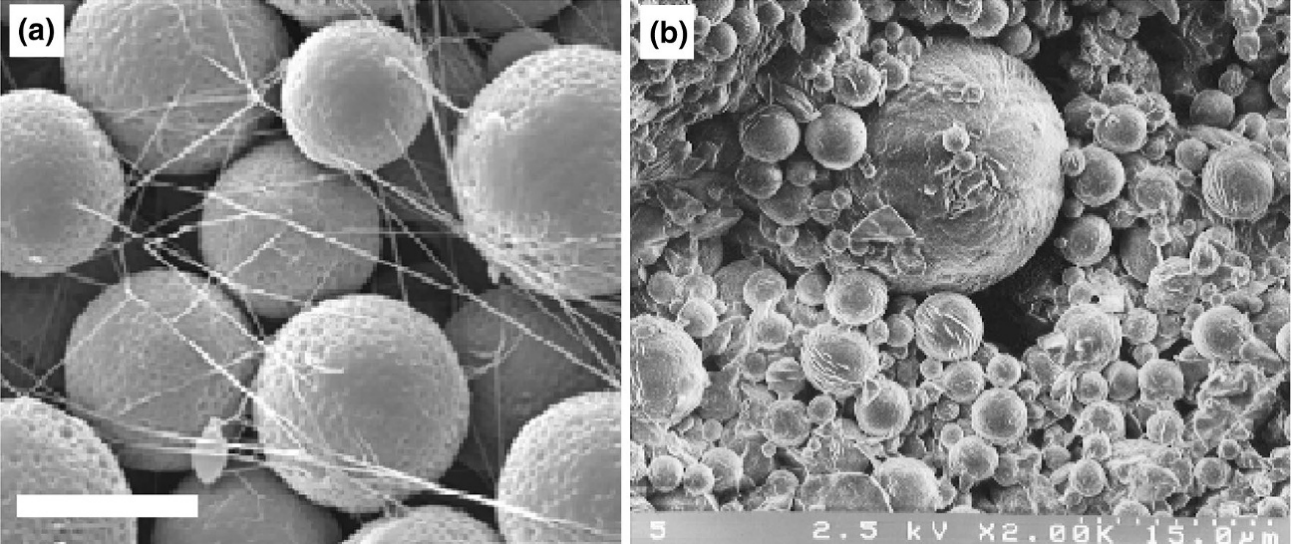
SEM images of **a** PCL microspheres produced using electrospraying process (Flow rate 0.2 mL/h, tip-to-collector distance 25 cm and voltage 16 kV). *Scale bar* represents 100 μ (Bock et al. 2011), and **b** PCL microspheres prepared using polymer blend melt technique (Lin et al. 1999)

Biodegradable PCL microspheres with diameters ranging from 10 to 20 μ with homogeneous embossed textures were produced (as presented in Fig. 13a) by Bock et al. (2011) via an electrospraying process. Briefly, PCL solutions (in chloroform and 5–10 wt % concentrations) were sprayed at 0.2 or 0.5 mL/h using a syringe pump at 10–18 kV and collected on aluminium foil at varying tip-to-collector distance (15–25 cm). They also investigated the biological effect of microspheres on the NIH3T3 cells using DNA quantification assays and direct contact methods and reported that no toxic residue was detected by this process, which suggested their suitability for further loading of bioactive components.

Solvent-free PCL microspheres (as presented in Fig. 13b) from PCL/PEG blends was developed by Lin et al. (1999); they transformed the molten polymer into microspheres (with diameters ranging from 1 to 20 μ) using rapid cooling in a freezer (−20 °C). As such, the toxicity associated with the organic solvent residue resulting from the conventional emulsion/solvent extraction process could be minimised.

#### PLGA microspheres

**Fig. 14:**
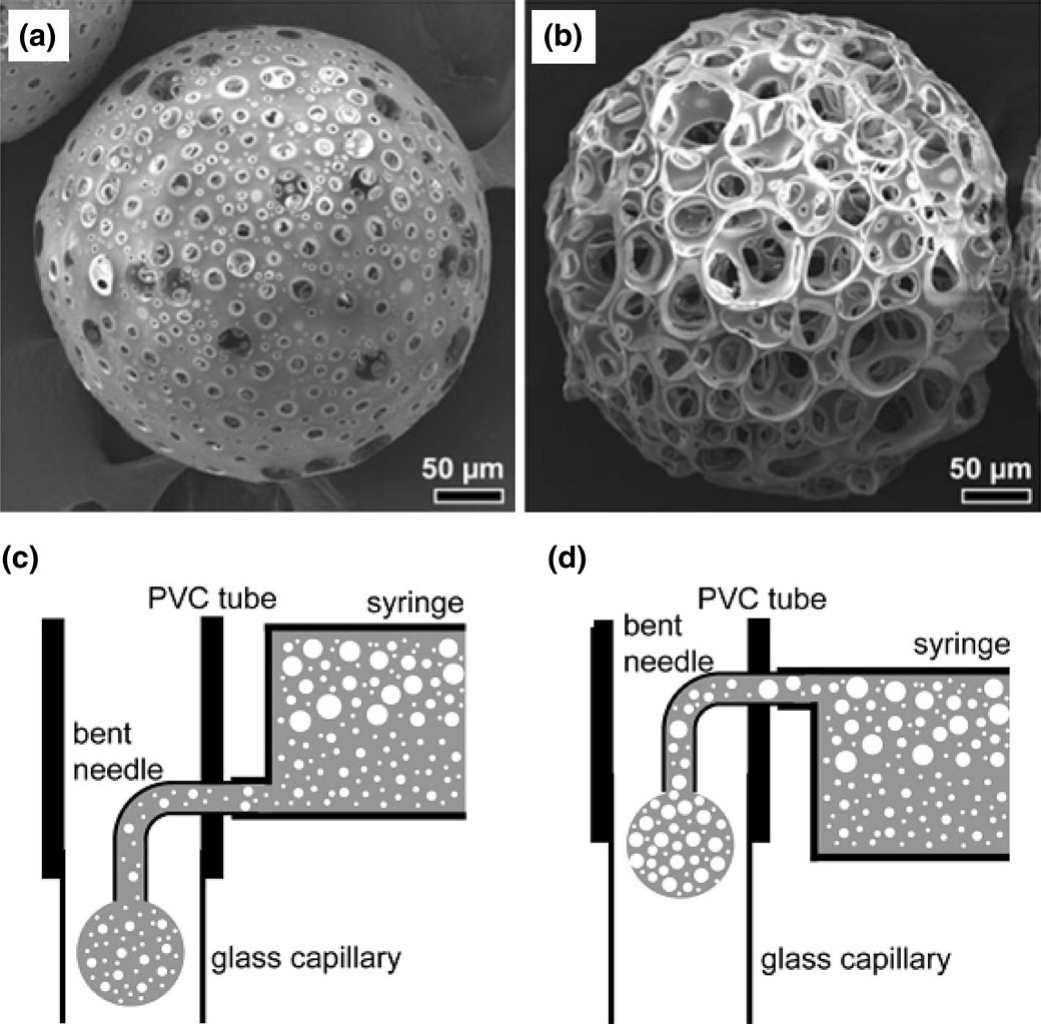
**a** SEM images of porous PLGA microspheres with small and **b** large pores (Choi et al. 2010)

Poly(d,l-lactide-co-glycolic acid) (PLGA), a copolymer of PLA and polyglycolic acid (PGA) has also been extensively used in the biomedical field for the synthesis of resorbable sutures, scaffolds, rods (Khorasani et al. 2008; Kohane et al. 2006; Morrow et al. 1974) and also porous microparticles (Oh et al. 2011; Ungaro et al. 2009; Wang et al. 2002; Yang et al. 2009). Porous PLGA microspheres with controllable pore size have been investigated by Choi et al. (2010). At first they produced a water-in-oil (W–O) emulsion by homogenising an aqueous solution of gelatine (7.5 wt %) and PVA (1 wt %) in a PLGA solution (2 wt % in dichloromethane). The W–O emulsion was then introduced into a fluidic device (fabricated using a glass capillary tube, needle and a poly(vinyl chloride) (PVC) tube), which transformed the phase into water-in-oil-in-water (W–O–W) droplets due to continuous flow of the aqueous phase (PVA solution). The resultant W–O–W droplets were subsequently solidified by solvent extraction and evaporation to generate porous microspheres (Fig. 14a, b). They also suggested that the pore size could be controlled using a fluidic device by arranging the syringe tip within the fluidic device. For example, when the syringe tip was placed at the bottom position during the phase transformation process, the W–O–W emulsion rich in small water droplets created microspheres with small pores due to the small size of the water droplets. On the other hand, microspheres with larger pore diameter were produced by placing the syringe tip at the upper level of the fluidic device.

#### Natural polymer microspheres

Chitosan (Dhawan and Singla 2003), alginate (Lemoine et al. 1998), collagen (Hong et al. 2012) and protein (Han et al. 2008) in the form of microspheres have been the most widely investigated natural polymers for use in the biomedical and tissue engineering fields. These natural polymers have the advantage that most of them are susceptible to biodegradation and are generally biocompatible.

**Fig. 15:**
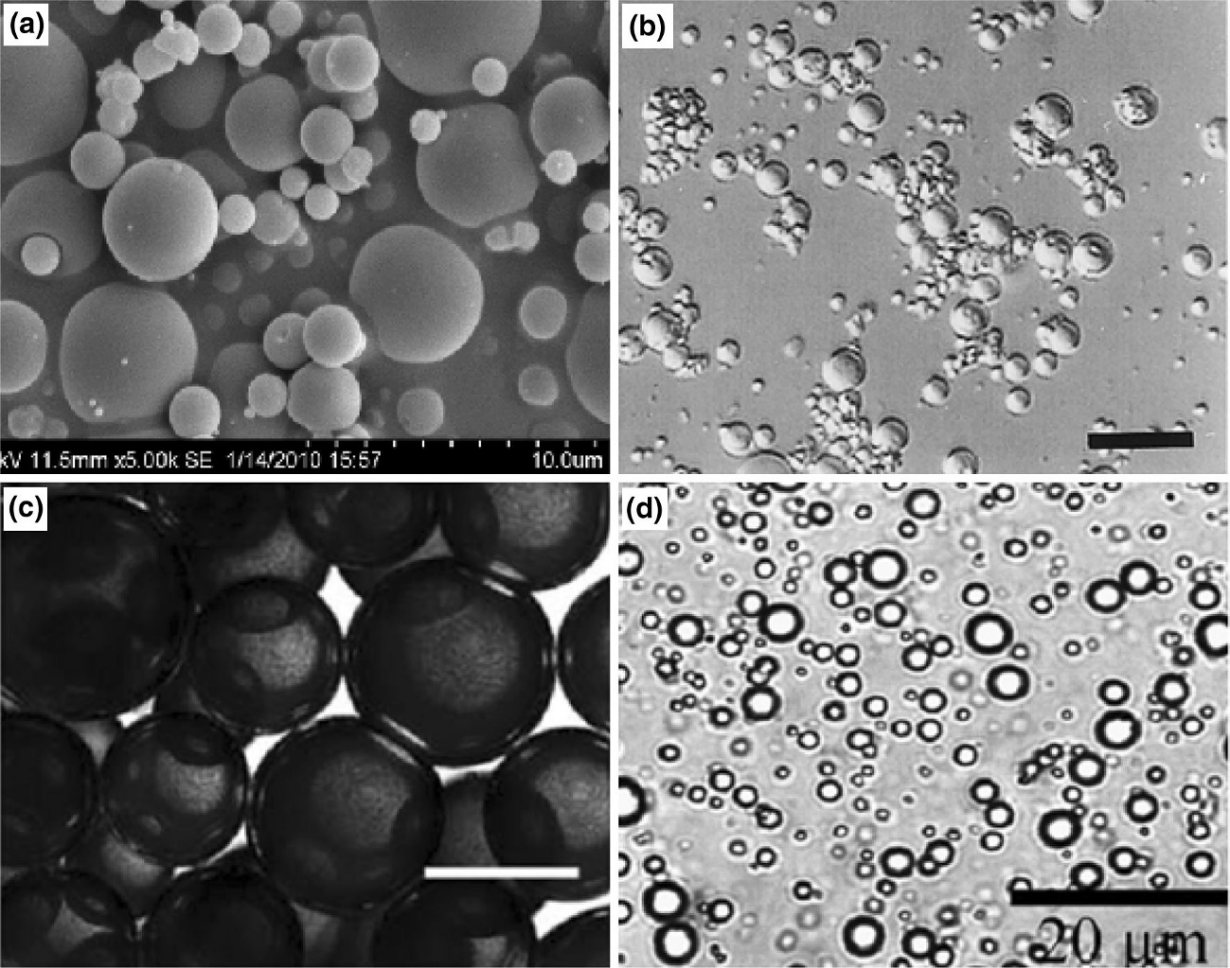
**a** SEM image of chitosan microspheres obtained by spray drying process Tao et al. (2013), **b** alginate microspheres prepared using emulsification technique (*scale bar* 62.5 µm) (Lemoine et al. 1998), **c** bright-field image of collagen microspheres in mineral oil produced via emulsification process (*scale bar* 200 µm) (Hong et al. 2012), and **d** protein microspheres prepared by ultrasonication (Han et al. 2008)

Chitosan, a naturally occurring biomacromolecular carbohydrate material, is widely employed for use in biomedical applications, such as tissue regeneration, bone void filling materials and as wound treatment due to its gel forming, self-hardening, bioadhesive, bacteriostatic and fungistatic properties (d’Ayala et al. 2008). Chitosan microspheres have been produced via spray drying (Oliveira et al. 2005; Torres et al. 2007), emulsification (Dhawan and Singla 2003), internal gelation (Akamatsu et al. 2010; Ribeiro et al. 2005), electrospinning and freeze drying processes. Dhawan and Singla (2003) investigated nifedipine-chitosan microspheres produced via an emulsification phase-separation process. They suggested that a high level of entrapment of nifedipine in the microspheres was achieved which exhibited excellent swelling properties. Porous structure within the microspheres could also be imparted by freezing the chitosan solution (in acetic acid) as a tiny droplet using liquid nitrogen followed by removal of solvent utilising freeze drying. Oliveira et al. (2005) produced chitosan microspheres by spraying chitosan solution (0.7 % w/v acetic acid solution) using a pressurised atomiser at around 125 °C. Water soluble chitosan microspheres (see Fig. 15a) investigated by Tao et al. (2013) and they were reported to be more effective in improving hyperlipidaemia in rats.

Alginates, derived from brown algae or soil bacteria (Baldwin and Kiick 2010) have also been investigated as gel-forming biomaterials for the treatment of oesophageal reflux, dermatology, wound healing and dental impression materials (Blaine 1947; d’Ayala et al. 2008). Alginate has also been fabricated into microspheres or microbeads utilising coagulation (Martinsen et al. 1989) and emulsification (Lemoine et al. 1998; Mofidi et al. 2000; Wan et al. 1992) processes. Commonly, microspheres have been formed by coagulating the alginate solution in the form of fine droplets using calcium chloride solution (Martinsen et al. 1989). However, a limiting factor highlighted for the coagulation method was its unsuitability for large-scale production. Mofidi et al. (2000) investigated the mass production of alginate microspheres using water-in-oil emulsion techniques (similar to production of the synthetic microspheres highlighted above). They stirred the alginate solution within a non-aqueous (oily phase) media in the presence of calcium chloride coagulant to form the microspheres. Non-aggregated alginate microspheres with an average diameter of 8 µm have been produced via an emulsification process (see Fig. 15b) by Lemoine et al. (1998). A high encapsulation efficiency (>90 %) and high loading (10 % w/w) of bovine serum albumin (BSA) within the alginate microspheres was achieved. They also reported the in vitro release profile of BSA which suggested a faster release rate of encapsulated BSA in PBS media. In addition, they also suggested that decrease of the release rate could be achieved by coating the alginate microspheres with poly(l-lysine).

Collagen is the most abundant insoluble fibrous protein found in extracellular matrix and in connective tissues like tendons, ligaments and skin. Similar to alginate and the synthetic polymers, collagen can also be fabricated into microspheres using the emulsification process (Nagai et al. 2010; Yao et al. 2013). Nagai et al. (2010) produced injectable collagen microspheres produced via a water-in-oil emulsion process followed by cross-linking with water-soluble carbodiimide. They also investigated the sustained release profile of recombinant human vascular endothelial growth factor (rhVEGF) from the collagen microspheres previously loaded with growth factor, which suggested that the sustained released rhVEGF remained bioactive during the culture period of 4 weeks. Apart from the conventional emulsification process for producing collagen microspheres as presented in Fig. 15c, Hong et al. (2012) developed a novel methodology for rapid production of collagen microspheres with encapsulated MDA 231 cells. A single chip comprising a microfluidic flow system was used to generate collagen micro-droplets, gelation and extraction processes of microspheres. At first, collagen micro-droplets were produced in aqueous and mineral oil phases and gelled immediately after their generation. The gelled microspheres were then extracted into a cell culture media where MDA 231 cells were incorporated within the microspheres, which suggested higher cell viability as well as larger number of microspheres recovery compared to the conventional centrifugation extraction process.

Proteins have been extensively investigated as drug carriers due their high biological activity, selective uptake by specific cells, non-antigenicity in denatured form and ability to provide multiple sites for the attachment of drug components. Proteins can also be combined with a wide range of drugs to generate derivatives with tailored pharmacological properties. Han et al. (2008) have prepared protein microspheres (see Fig. 15d) with an average diameter of 1 µm by sonicating silicon oil in an aqueous solution of protein. They have also investigated the loading of red dye into the microspheres and suggested that drugs can be incorporated within the microspheres by simply dissolving drugs into the oil phase prior to sonication. Spherical shaped protein molecules have also been produced by dispersing aqueous proteins into mineral oil and subsequently polymerised using glutaraldehyde (Bergquist et al. 1972). However, reproducibility of this protein microspheres production was suggested to be dependent on several parameters, which includes the mode of dispersing proteins into oil, absolute amounts of constituents and pH of the reaction. They have also investigated the Immunological activity of the protein microspheres produced and it was found that they remained practically unaltered after multiple freezing and thawing and also after several weeks of storage at −70 °C.

## Use of microspheres for biomedical applications

One of the most widely prevailing applications for microsphere use is as a drug delivery vehicle. By specifically selecting biocompatible materials, tailoring the physical structure (i.e. inclusion of interconnected pores for example) and selecting a convenient method of drug incorporation (e.g. incorporation during or after synthesis/fabrication), it is possible to control the rate of drug release. In particular, microspheres are hugely advantageous for encapsulation of fragile drugs such as nucleic acids and proteins (Berkland et al. 2004; Kim and Pack 2006; Xia et al. 2013) by providing protection for biological entities that would otherwise be rapidly destroyed by the body. Other applications have included use of microspheres as controlled release vehicles for vaccines, since their spherical shapes are ideal for take up by antigen-presenting cells. The vast majority of materials used to fabricate these spheres for such applications are biopolymers such as PLA, PLGA and PCL (Freiberg and Zhu 2004; Kim and Pack 2006).

The high sphericity of these particles has also shown desirable attachment of cells and is thought to improve delivery to the body via injection, as well as reduce inflammatory responses associated with foreign body implantation. Such cells recently investigated include Saos-2 cells (Perez et al. 2011), OCT-1 osteoblast-like cells (Hu et al. 2014), neural cells (Lin et al. 2014), chondrocytes (Chen et al. 2006) and stem cells (Perez et al. 2014), to mention a few.

Creating porous microspheres has enabled these structures to be used as tissue regeneration scaffolds, since high interconnectivity enables the cells to seed more efficiently throughout the structure, as well as providing a large volume and surface area for nutrient transport/waste removal, and ultimately cell proliferation and differentiation (Cai et al. 2013).

The large surface area, porosity and volume associated with microspheres make them an ideal candidate to act as carriers for biological components such as growth factors, hormones, therapeutic agents, etc. directly to the target site (Cai et al. 2013). A number of products based on ceramic and polymer microspheres (see Table 1) have already made it to Market with potential use with pharmaceuticals, biomedical and tissue engineering sectors.

**Table 1:** List of commercial products containing ceramic and polymer microspheres currently available

Product name	Microsphere Materials/loaded *drugs or biological components*	Application	Company name
Cerasorb^®^	β-TCP	Dentistry	Curasan
Hydros	Brushite calcium phosphate	Orthopaedics	Biomatlante
Calcibon^®^	HA (precipitated)	Orthopaedics	Biomet
Lupron Depot	PLGA loaded with *Leuprolide acetate*	Drug delivery	TAP Pharmaceutical Products Inc.
Nutropin Depot	PLGA loaded with *Recombinant human growth hormone*	Growth hormone regulator	Genetech, Inc.
Enantone LP	PLGA loaded with *Leuprorelin*	Treatment of prostate cancer	Takeda Pharmaceutical Company Limited
Somatulin LP	PLGA loaded with *Lanreotide*	Treatment of acromegaly	IPSEN pharma
Sandostatin LAR	PLGA loaded with *Ocreotide*	Treatment of acromegaly	Novartis
Cytodex 3	Collagen (denatured) cross-linked with *dextran*	Microcarriers for various cell lines (tissue engineering)	GE healthcare
Cultisphere^®^	Getalin	Microcarriers for various cell lines (tissue engineering)	Percell Biolytical

Sources: product information has been collected from the respective company’s website

Over recent years much work has been conducted with the aim to develop and improve the use of microspheres for biomedical applications. Therefore, due to rapid increase in scientific research in this field it is expected that new commercial products with more specific features and functionality will continue to be developed.

## Challenges and future prospects for microspheres

Microsphere production methods developed over the years have resulted in favourable yields of microspheres in terms of size and sphericity which also happen to be material specific. However, it is apparent that some challenges still remain, the most prominent of which include difficulties encountered with large-scale production of microspheres. Many of the manufacturing processes utilised involve several steps, particularly those fabricated from polymers and ceramics, making it more difficult to scale up the process due to cost and time. The numerous steps involved with fabricating microspheres can also potentially alter the properties of the material once spheroidised, as is the case for silicate-based glasses, where thermal processing can cause crystallisation of the glass. This in turn can affect the properties of the glass microspheres such as increasing their brittleness and alter their degradation profiles. Ceramics can also have brittle characteristics as well as having high production costs and lengthy and/or complicated manufacturing processes. In addition, achieving specific control over alternate geometrical features (such as size, shape, yield and reproducibility) will be the key.

As demand rises for production efficiency, especially for materials with enhanced properties, microsphere production will hopefully rise to the challenge as they hold superior structural properties related to other irregular shaped particle morphologies. Microspheres are beginning to look more promising for use in biomedical applications with several companies already exploiting these in the pharmaceutical and health care industry [for example, MoSci Corporation (USA) and Locate Therapeutics (UK)].

## Summary

This article aimed to review the different methods employed to produce microspheres from various kinds of materials including glass, ceramics and polymers. Production of these microspheres is material dependent; however, a majority of the fabrication methods used tend to be quite lengthy and can take several days to prepare, in most cases requiring several steps for fabrication. For example, various methods, such as passing ground glass particles down a vertical tube furnace, sol–gel and spray drying of sols and flame spheroidisation processes, were identified to produce glass microspheres. In case of CaP based ceramic microspheres production precipitation, spray pyrolysis, electrospraying, emulsification processes were employed with a broad range of properties. Other methods, such as emulsion-solvent evaporation, spray drying, electro-spinning, gelation followed by emulsification, suspension polymerisation and ultrasonication processes were discussed to produce polymer microspheres.

As a result of the diversity of microspheres produced employing various materials, a broad range of properties can be obtained. For example, materials composition, particle size distribution, degradation rate, adsorption and desorption kinetics and porosity will be key for successful use of the microspheres for biomedical applications. Various methods have been identified to control the size distribution in a narrow range, such as initial particle size monitoring (in case of glass microspheres), precipitation and/or coagulation reactions as well as stirring time (for ceramic microspheres), ratio of water and oily phases and their blending speed (for polymer microspheres). Degradation, release of bioactive components as well as resorbability of the materials used for microsphere production are also key properties depending on their target medical applications. Degradation rates can be controlled by altering the compositions (for glasses) and also blending with some types of hydrophilic materials. However, ceramic-based microspheres pose slow resorption rates compared to the glass and polymer microspheres. Porosity is another very important property of these microspheres, especially for drug delivery and tissue engineering applications. Porous microspheres can provide higher loading efficiency, adequate transportation of nutrients and further control over the release behaviour of drugs, growth factors and other biological components. They are favourable for cell attachment and proliferation due to their larger surface area. In addition, porous microspheres can protect cells encapsulated within the pores from physical damage during the material handling and delivery processes employed. In addition, other features such as interconnected and open porosity, favourable pore size and appropriate mechanical properties need to be considered during porous microsphere production. Furthermore, despite the superior properties of porous microspheres, non-porous microspheres can have advantageous features for some specific applications, such as bone regeneration (in case of load bearing applications) where higher mechanical properties are required. In addition to this, precise control over pore size has been found to be difficult; hence manufacturing technologies need to be improved to create reproducible porosity and pore sizes. Furthermore, although considerable efforts have been made in limiting the initial degradation and burst release profiles on implantation within the body, these areas still warrant further research and application.
